# Cortico-Hippocampal Oscillations Are Associated With the Developmental Onset of Hippocampal-Dependent Memory

**DOI:** 10.3389/fnins.2022.891523

**Published:** 2022-06-23

**Authors:** María A. García-Pérez, Martin Irani, Vicente Tiznado, Tamara Bustamante, Marion Inostroza, Pedro E. Maldonado, José L. Valdés

**Affiliations:** ^1^Departamento de Neurociencia, Facultad de Medicina, Universidad de Chile, Santiago, Chile; ^2^Departamento de Psiquiatría, Centro Interdisciplinario de Neurociencias UC, Pontificia Universidad Católica de Chile, Santiago, Chile; ^3^Biomedical Neuroscience Institute (BNI), Facultad de Medicina, Universidad de Chile, Santiago, Chile; ^4^Institute of Medical Psychology and Behavioral Neurobiology, University of Tübingen, Tübingen, Germany; ^5^National Center for Artificial Intelligence, CENIA, Santiago, Chile

**Keywords:** hippocampus, postnatal, memory, sleep, electrophysiology

## Abstract

Hippocampal-dependent memories emerge late during postnatal development, aligning with hippocampal maturation. During sleep, the two-stage memory formation model states that through hippocampal-neocortical interactions, cortical slow-oscillations (SO), thalamocortical Spindles, and hippocampal sharp-wave ripples (SWR) are synchronized, allowing for the consolidation of hippocampal-dependent memories. However, evidence supporting this hypothesis during development is still lacking. Therefore, we performed successive object-in-place tests during a window of memory emergence and recorded *in vivo* the occurrence of SO, Spindles, and SWR during sleep, immediately after the memory encoding stage of the task. We found that hippocampal-dependent memory emerges at the end of the 4th postnatal week independently of task overtraining. Furthermore, we observed that those animals with better performance in the memory task had increased Spindle density and duration and lower density of SWR. Moreover, we observed changes in the SO-Spindle and Spindle-SWR temporal-coupling during this developmental period. Our results provide new evidence for the onset of hippocampal-dependent memory and its relationship to the oscillatory phenomenon occurring during sleep that helps us understand how memory consolidation models fit into the early stages of postnatal development.

## Introduction

Hippocampal-dependent memories are not innate (Westbrook et al., 2014; Ramsaran et al., 2016, 2019; Contreras et al., 2019), and their postnatal expression has remained controversial. The appearance of episodic memory has been closely related to the maturation of the hippocampus (Jabès et al., 2011; Farooq and Dragoi, 2019; Muessig et al., 2019; Nadel, 2021) and its role in spatial navigation, including place cells, grid cells, head direction cells, among others, providing all the spatial information that the animal would need (O’Keefe and Dostrovsky, 1971; Taube et al., 1990; Sargolini et al., 2006; Ainge and Langston, 2012). The first signs of hippocampal-dependent memory have been reported to emerge as early as postnatal day 17 (P17) (Westbrook et al., 2014); however, it is not fully expressed until late adolescence of the rat (i.e., postnatal weeks 4–6) (Westbrook et al., 2014; Contreras et al., 2019). Since episodic memory uses spatial and temporal resources, there is a diversity of task parameters to be considered. From the literature, during the transition from juvenile to adolescent (P25–P48), rats acquire the ability to discriminate a hippocampal-dependent memory task (Westbrook et al., 2014; Ramsaran et al., 2016, 2019; Contreras et al., 2019).

In adult rats, sleep is necessary for memory consolidation (Rasch and Born, 2013). The “two-stage” (Marr, 1971; Buzsáki, 1989) and the “active system consolidation” (Marr, 1971; Buzsáki, 1989; McClelland et al., 1995; Frankland and Bontempi, 2005; Rasch and Born, 2007) models propose that the consolidation of hippocampal-dependent memories occurs through a cortico-hippocampal dialog where sleep oscillations play a critical role. In particular, the temporal coupling between the cortical slow-oscillations (SO, 0.5–4 Hz), thalamocortical Spindles (9–16 Hz) (Klinzing et al., 2016; Hahn et al., 2020; Silversmith et al., 2020), and hippocampal sharp-wave-ripples (SWRs, 100–250 Hz) (Siapas and Wilson, 1998; Mölle et al., 2006; Peyrache et al., 2011; Staresina et al., 2015) are known to have a causal role in memory consolidation (Girardeau et al., 2009; Maingret et al., 2016; Latchoumane et al., 2017). These three-cardinal sleep oscillations have also matured during postnatal development (Buhl and Buzsáki, 2005; Olini et al., 2013; Lindemann et al., 2016). However, it has not been fully addressed how memory consolidation fits with the postnatal emergence of these oscillations and the temporal coupling between them.

We conducted a hippocampal-dependent recognition memory test in conjunction with cortical and hippocampal electrophysiological recordings *in vivo* across several postnatal days in rats to explore this mechanism. This experimental set-up allowed us to determine when hippocampal-dependent memory emerges during postnatal development and to explore how SO, Spindles, and SWRs correlate with this cognitive capacity. Our data indicate that hippocampal-dependent memory emerges by P32, with task performance equivalent to adult rats, and is not a result of early task overtraining. In addition, the *in vivo* electrophysiology revealed developmental changes in the three central oscillations associated with memory consolidation during sleep on features such as density, frequency, duration, and temporal coupling.

## Materials and Methods

### Animals

Forty male Long-Evans rats, born and bred at the Centro de Investigaciones Médicas (CIM) of the Pontificia Universidad Católica de Chile facilities, were used in this study. All pups were kept with their mother until weaning (P19). Animals were housed with free access to water and food in a controlled environment room with a temperature of 22 ± 1°C and light/dark cycles of 12/12 h, ZT0 = 8:00 AM. Rats were grouped by age for the object place recognition (OPR) task at different stages of development: juvenile (P26–28, *n* = 12); these same animals at peri-adolescent stage (P30–32, *n* = 11 due to a lost electrode implant); young adult rats (P90, *n* = 17). Finally, a different group at P32 rats (*n* = 11) underwent the OPR test without previous repetitions of the task (P32-NoRep group). All surgical and experimental procedures were conducted under the National Institute of Health (USA) Guide for the Care and Use of Laboratory Animals (NIH Publications No. 80–23, revised 1996) under the approval of our institutional Biosafety and Ethical Committee of the Universidad de Chile (CBA #1108, FMUCH) and Pontificia Universidad Católica de Chile (CEC-CAA ID:180430001). All efforts were made to minimize suffering and the number of animals used.

### Animals Groups

The 40 rats included in this study came from 7 different litters (2–5 rats per litter) divided into three groups. The first group was tested in the Object-Place Recognition (OPR) memory task from postnatal days P26 to P32 (*n* = 12, four litters); four of these animals (coming from two different litters) received implants for electrophysiological recording. Three out of four of these animals reached adult-like performance by P32. A second group, P32-NoRep (*n* = 11; five litters), was included to test for overtraining effects, and the third group of young adult rats was tested in the OPR task at P90 (*n* = 17, seven litters.

### Object-Place Recognition Task

Rats were handled daily for 10 min over five consecutive days. Before the memory test, the rats were habituated to the arena for 2 days. During the first day, the animals were allowed to freely explore the arena in groups of no more than four rats for 20 min. Immediately after, all rats were allowed to rest for 30 min on top of a flowerpot containing some bedding materials. On the second day, the same group of rats explored the empty test arena for 10 min, and subsequently, all animals individually explored the test arena with one object for an additional 10 min. Then the rats were placed on top of a flowerpot to rest under the same conditions as the previous day. This habituation procedure was repeated twice a day (am/pm); after each session, the rats received gentle handling for 10 min.

The OPR task consisted of an encoding and a recall phase, separated by a 3-h memory consolidation interval. Only data from rats that slept during the memory consolidation phase was used to assess memory performance and sleep oscillations. Sleep was determined by visual inspection of video recording of each animal and then confirmed by sleep oscillation analysis. First, the rats could explore two identical objects in adjacent corners of the arena (encoding phase). Then one of the objects was moved to the opposite corner in a new location, while the other remained at the same location (recall phase). In each test, the rat had 5 min to explore the arena. White noise was played at a constant intensity (60 dB) during all procedures to environmental sounds. The test arena consisted of a dark gray square made of gray PVC and was illuminated with indirect light. Two different sizes were used depending on the age of the rats, as has been suggested before (Reger et al., 2009): a small arena for rats between P26 and 32 (40 cm × 40 cm × 40 cm) and a larger arena for rats at P90 (80 cm × 80 cm × 40 cm). A series of distal cues were available to facilitate spatial location in the room. In addition, the entrances to the arena were randomized for each rat to avoid using the entry position as a proximal cue between the encoding and recall phases. Objects for exploration were glass bottles of different shapes (height: 10–18 cm and 22–29 cm for rats between P26–32 and P90, respectively).

The bottles were placed 10–15 cm equidistant from the walls to avoid the preference of the animals to stay in corners. The trial was discarded if the rats expressed a spontaneous preference for one of the two objects during the encoding phase. After each test, the objects and the open field were thoroughly cleaned with a 70% ethanol solution. The behavior of the rats was videotaped during the experiment with an overhead camera. The animal’s path in the arena was reconstructed using the open-source video tracking system IdTracker (Pérez-Escudero et al., 2014), and the offline quantification of movement was conducted using a custom-made MATLAB routine (Mathworks, Inc.).

### Analysis of Memory Performance

Exploration was defined as whenever a rat had its nose pointing toward an object no more than 2 cm from the object. Climbing on an object or sitting next to them without any sign of active exploration were not scored. The memory index was calculated as the difference in time spent exploring the displaced object and non-displaced object with respect to the total time of exploration of both objects: (displaced object – non-displaced object)/(displaced object + non-displaced object). A value close to 1 indicates higher exploration of the displaced object, while a value close to −1 indicates that the animals prefer the non-displaced object over the displaced one. A value close to 0 indicates no preference. The rats who explored at least 1 s of both objects during the encoding phase of the task were considered for analysis. Total distance traveled during the encoding and recall phases was used as an index of motor activity.

### Surgical Procedure and Implants

On P20, rats (*n* = 4) were anesthetized with isoflurane (3% induction, 1–2% maintenance) and fixed in a stereotaxic frame. Body core temperature was kept at 37°C using a heating pad. Animals were implanted with a custom-built microdrive consisting of 4 independent movable tetrodes grouped in two separated bundles made of polyamide tubes (ID = 0.343 mm, Masterflex Transfer Tubing, United States) and positioned over the right hemisphere. Each tetrode was loaded into a stainless-steel guide tube (27 G; Components Supply Co, FL, United States), allowing tetrodes to move independently. A single craniotomy was performed, and the microdrive was implanted with tetrodes distributed according to the following coordinates: one tetrode targeting the medial parietal association cortex (MPtA), anteroposterior −3.5 mm; mediolateral 2.6 mm; and 1.5 mm in dept, and three tetrodes targeting the dorsal CA1 region of the hippocampus (−3.5 mm anteroposterior; 2.6 mm mediolateral and 2.5 mm in depth). All relative to bregma and adjusted to skull size at P20 in agreement with the rat brain atlas (Paxinos and Watson, 2007). The microdrive was anchored to the skull with 2–4 stainless steel jewelry screws and dental acrylic. A teflon-coated wire (stainless steel wire, 0.003”) was welded to two of the screws in the skull above the cerebellum and used as ground. Antibiotics (Enrofloxacin 5%, 10 mg/kg i.p) and anti-inflammatories (Ketophen 0.2 mg/kg i.p) were administered at the end of surgery and for three consecutive days before performing any experimental procedure. During the recovery time, rats received food and water *ad libitum*, and general health was monitored.

### Electrophysiological Recordings and Processing

Electrophysiological signals were simultaneously recorded from each of the 4 × 4 = 16 wires. Tetrodes were made of four twisted 17 μm nichrome wires (AM Systems; WA, United States), gold plated to an impedance of ∼1 MΩ. Each tetrode was independently lowered to the desired region at a speed of no more than 300 μm/day from days P21 to P26 to optimize signal yield and stability. The leads of the tetrodes were connected to a unity-gain head stage, and all the data were collected using an RHD2000 Recording System (Intan Tech, CA, United States). Neural signals were amplified (200-fold), digitized at 20 kHz, and filtered between 0.5 and 450 Hz. Local Field Potential (LFPs) were derived from wideband signals by downsampling all channels to 1,000 Hz. Recordings were obtained while the animal was resting on top of a flowerpot, which was used to prompt spontaneous sleep and reduce movement. Each animal was recorded every 2 days, from P26 to P32. All recording sessions were done simultaneously, from 09:00 a.m. to 14:00 h (ZT1 to ZT6). To classify sleep stages, each LFP channel signal was z-scored and segmented into non-overlapping 5 s windows during episodes by which the animals were immobile. The power spectral density was computed and averaged for cortical delta/SO (0.5–4 Hz) and hippocampal theta (4–8 Hz) frequency bands for each time window. Then, a k-means classifier was employed to discriminate between NREM sleep and REM/awake states (Maingret et al., 2016; Silversmith et al., 2020), Supplementary Figures 2c,d. Only long sleep periods (>30 s and five consecutive windows) were analyzed. The selected windows were further confirmed by visual inspection.

### Analysis of Local Field Potential Recordings

All the animals considered for Sleep oscillation analysis were tested in the OPR task from P26 to P32. In addition, the analysis of sleep oscillation across development ages was extended from P26 to P42 to determine if any changes associated with age persisted even after P32, which was the critical day when memory capacities emerged. Three rats were recorded from P26 to P42. Two of them reach adult like memory performance by P32, while the third one did not. The fourth rat was recorded during sleep from P26 to P31 because it’s lost the electrodes connectors, however this animal showed adult like memory performance by P32 and was included in the rest of sleep oscillation analysis.

#### Oscillatory Event Detection

The normalized LFP signal recorded from the MPtA during NREM sleep was filtered between 0.5 and 4 Hz for offline SO detection. SO were identified according to the following criteria (adapted from (Mölle et al., 2006; Durán et al., 2018)): (i) two consecutive negative-to-positive crossings of the signal occurred within 0.2–1.0 s; (ii) the peak-to-trough amplitude of each event was lower than the mean of the whole signal plus 3 SD and higher than the mean minus 6 SD.; and (iii) the trough amplitude was lower than the mean of the whole signal minus 1.5 SD and higher than the mean minus 4 SD. SO onset was considered aligned to the downstate, as reported before (Mölle et al., 2011; Klinzing et al., 2016).

The LFP signal from MPtA was also band-pass filtered between 9 and 17 Hz for Spindles detection, then a smoothed envelope of the signal was obtained by calculating the magnitude of the Hilbert transform and convolving it with a Gaussian kernel (α = 2.0). Subsequently, two thresholds for Spindle detection were determined based on the mean and SD of the whole envelope during NREM sleep (lower: 1 SD; upper: 2 SD). Events in which the envelope exceeded the thresholds for at least 500 ms were considered Spindles. Finally, Spindles separated by 300 ms or less were merged as in previous reports (Maingret et al., 2016; Silversmith et al., 2020).

For SWR detection, the normalized LFP signal recorded in the dorsal CA1 region of the hippocampus was band-pass filtered between 150 and 250 Hz. The squared magnitude of its Hilbert transform was calculated to improve the detection of small amplitude SWRs, according to Maingret et al. (2016). SWRs were defined as events for which the amplitude was at least 2 SD above the mean power envelope of the signal, and the peak of the SWR signal was higher than 5SD, and of a duration from 30 to 100 ms (Maingret et al., 2016).

#### Sleep Oscillation Coupling Analysis

For SO and Spindle coupling, the down-states of the SOs and the beginning of the Spindles were used to generate a cross-correlation analysis to detect coupled events in a temporal window of ± 500 ms. The coupled SO-Spindles recorded per session for each rat were analyzed as total events during each sleep period throughout postnatal development (P26 to P42). In addition, only coupled events that surpassed the mean ± 2SD of the 1000 permutations of Spindles and SO time stamps (MonteCarlo analysis) were considered significant.

For SO-SWR coupling, the valley of SO and SWR were used as a timestamp to generate a cross-correlation between those signals. Then SRWs nested on the valley of a SO in a time window of ± 500 ms were detected. Finally, coupled events were quantified for each rat during each period of sleep (P26–P42). Events that exceeded the mean ± 2 SD of 1000 permutations of SO and SWR timestamps (MonteCarlo analysis) were considered significant.

For Spindle-SWR coupling, the peak amplitude of the Spindle and the maximal trough amplitude of the SWR were used to generate a cross-correlation between those signals to detect coupled events, utilizing a ± 500 ms window. Coupled events were quantified for each rat during each period of sleep (P26–P42). Events that exceeded the mean ± 2 SD of 1000 permutations of the SWR and Spindle timestamps (MonteCarlo analysis) were considered significant.

#### Phase Coupling

Phase coupling between SO and Spindles and SO and SWR was calculated at each postnatal age (P26–P42). For this purpose, the LFP was filtered as was described before. Then using the maximum peak amplitude of the Spindle or the maximal trough amplitude for SWR (valley) as event timestamps, we computed the phase angle of the Spindle or SWR events relative to the SO using a Hilbert transform according to Hahn et al. (2020), the mean direction of the phase angles (i.e., preferred phase) and coupling strength were determined using CircStat Toolbox functions (circ_mean, and circ_r; Berens, 2022). Finally, the non-uniformity of the resulting vector was tested with the Rayleigh test using the function circ_rtest (Berens, 2022).

#### Time-Frequency Analysis

A multitaper frequency analysis was performed for each SO event in the frequency range of Spindles and SWRs, looking at the time window + 1 s around the timestamp of the second downstate of each SO. The baseline period was considered −4.5 to −2 s before an SO was detected. This approach allowed us to normalize each time window. The big SO average was plotted as a visual reference guide aligned at 0 in the down-state. Statistical comparisons were performed by a non-parametric cluster-based permutation test (MonteCarlo, *n* = 1000), comparing the power in every frequency band in the time window of interest period (−1 to 1 s, from down-state of SO) with respect to the corresponding power in the baseline period. Each SO event was locked at 0 lag in the downstate of the SO. We compared for changes in power in the Spindle frequency range (5–20 Hz) using a cluster alpha of 0.02, critical alpha 0.005 two-sided, and plotted with a significance mask of *p* < 0.01; and for changes in SWR frequency range power (100–300 Hz) using a cluster alpha of 0.05, critical alpha 0.025 two-sided, and plotted with a significance mask of *p* < 0.05.

### Histology

At the end of the experiment, electrolytic lesions were made at the recording sites passing 50 μA of current for 10 s through one lead of each tetrode. The next day, animals were deeply anesthetized with 4% isoflurane and transcardially perfused with 300 mL of saline solution (0.9% NaCl) and 400 mL of 4% paraformaldehyde in phosphate buffer (PB 0.1 M pH 7.4). The brain was carefully removed from the skull and post-fixed in the same fixative overnight and then transferred to a solution of 30% sucrose in PBS (PB 0.01 M NaCl 0.9%) with 0.02% sodium azide. Three days later, the brains were blocked in the coronal plane and subsequently cut into slices of a thickness of 80 μm with a vibratome (World Precision Instruments, Sarasota, FL, United States) in ice-cold PBS. The tissue was then processed for Nissl staining to confirm the correct location of the recording tetrodes. We confirmed the position of three electrodes in CA1 and one electrode in CA3 (see Supplementary Figure 2a,b). Even though some differences in the SWR detection were observed, the occurrence of SWR for each animal across days was not different. See Supplementary Table 1.

### Statistical Analysis

All statistical analyses were performed using SigmaPlot (Systat Software Inc.) and custom-made scripts from MATLAB (Mathworks, Inc.). *Behavioral analyses.* The non-parametric two-sided Wilcoxon signed-rank test was used to compare the discrimination index for each rat on each postnatal day with respect to chance. A two-sided Wilcoxon rank-sum test was used to compare groups (i.e., juvenile vs. adults). For discrimination index analysis, normality was determined by using the Kolmogorov-Smirnov test. Group differences between postnatal days were detected using a one-way repeated ANOVA followed by Bonferroni’s *post-hoc* test for multiple comparisons (control group: P26). The traveled distance was analyzed using a one-way repeated ANOVA and total object exploration using the Friedman repeated-measures ANOVA on ranks test. Data are reported as mean ± SEM. The statistical level of significance was *p* < 0.05. For correlational analyses, we calculated linear regressions by Spearman rank correlations. For effect sizes, squared Spearman correlation coefficients (rho) and *p*-values are reported.

## Results

### Postnatal Hippocampal-Dependent Memory Onset

**FIGURE 1:**
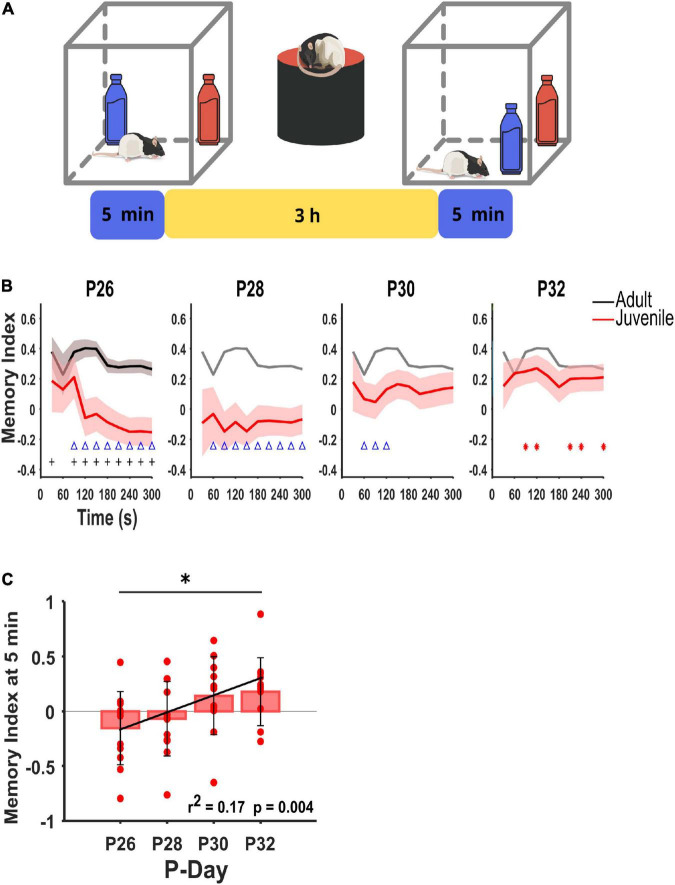
Hippocampal-dependent memory onset. **(A)** Experimental design. Rats were tested on an OPR task on different postnatal days (P26, P28, P30, P32) or in adulthood (P90). Each animal explored the test arena with two identical objects for 5 min during the encoding and recall phases, with a retention phase of 3 h, where the animal rested on top of a flowerpot. One of the objects was moved to a novel location during the recall phase, while the other remained in the same place. Rat figures were obtained from the GitHub repository (Eackermann, 2018). **(B)** Average memory index (Mean ± SEM) during the recall phase throughout postnatal days. The memory indexes are shown for the entire task duration (5 min, 30 s/bin). The black line indicates adult rats (P90), then the same line was repeated in gray for referencing the following P-days. Juvenile subjects (P26–32 are indicated by the line in red). The P90 group showed significant differences against chance level (zero memory index); they were only shown at P26 as reference (” + “; *p* < 0.034, two-sided Wilcoxon signed-rank). Only P32 juvenile rats showed a significant memory index against chance level (*p* < 0.042, two-sided Wilcoxon-signed-rank, dot in red). P26–P30 rats showed non-significant differences concerning chance (*p* > 0.083, two-sided Wilcoxon rank-sum). Only at P32 juvenile rats show no significant difference with respect to adults (*p* > 0.082, two-sided Wilcoxon rank-sum, triangle in blue). P26 and P28, *n* = 12; P30 and P32 *n* = 11. **(C)** Average (Mean ± SEM) of the cumulative memory index at 5 min for each postnatal day (P26, P28, P30, and P32). The memory index becomes positive and significant only at P32 [*p = 0.043, one-way repeated ANOVA, *F*_(3,11)_ = 3.057], followed by the Bonferroni’s *post-hoc* test, *p* = 0,038 between P26 and P32. Linear regression analysis between the OPR cumulative time (5 min) against P-days indicate a positive and significant correlation (*r*^2^ = 0.170; *p* = 0.004).

We examined the emergence of the hippocampal-dependent memory between P26 and P32 with successive exposures to the OPR task (Figure 1A) with a 3-h retention interval. Memory performance was also evaluated at P90 to compare our results with adults with fully developed hippocampal-dependent memory consolidation. We observed that the memory index improved with age and reached adult-like performance by P32 (Figure 1B).

Rodents from P26 to P30 exhibited no OPR memory as no preference between objects in the recall phase was observed (*p* > 0.0830), and significant differences with respect to the adult group memory index Figure 1B (*p* < 0.0431, blue triangles). Interestingly, by P30, the memory index increased significantly, but during the first 2 min of the task (*p* < 0.0431, blue triangles). Notably, by P32, the adolescent rats successfully distinguished the displaced object from the non-displaced object after the 3-h retention period (*p* < 0.042, red dots), reaching the adult-like memory index during the entire task. In addition, we also determined the changes in the mean memory index during the total task time (5 min) for each postnatal day; as has been reported before, and we could confirm that from P26 to P30, the animals showed no preference with a memory index close to 0 during the recall phase (Figure 1C). However, the memory index becomes positive and significant at P32 [*p* = 0.043, *F*_(3,11)_ = 3.057], followed by multiple comparisons vs. P26 (*p* = 0.038, black asterisk; Figure 1C), indicating that rats have a marked preference for exploring the displaced object over the non-displaced one. A progressive improvement in memory capacities could be observed across days since the linear regression analysis indicated a positive and significant correlation between memory index and postnatal days (*r*^2^ = 0.170; *p* = 0.004; Figure 1C). Our results suggest that only by P32 are the rats able to achieve a memory index similar to an adult (Figures 1B,C). However, this capacity does appear to be developing across this postnatal period, with P32 as a critical developmental timespoints for the emergence of hippocampal-dependent spatial memory consolidation.

### The Developmental Onset of Spatial Memory Consolidation Is Not Due to Task Overtraining

**FIGURE 2:**
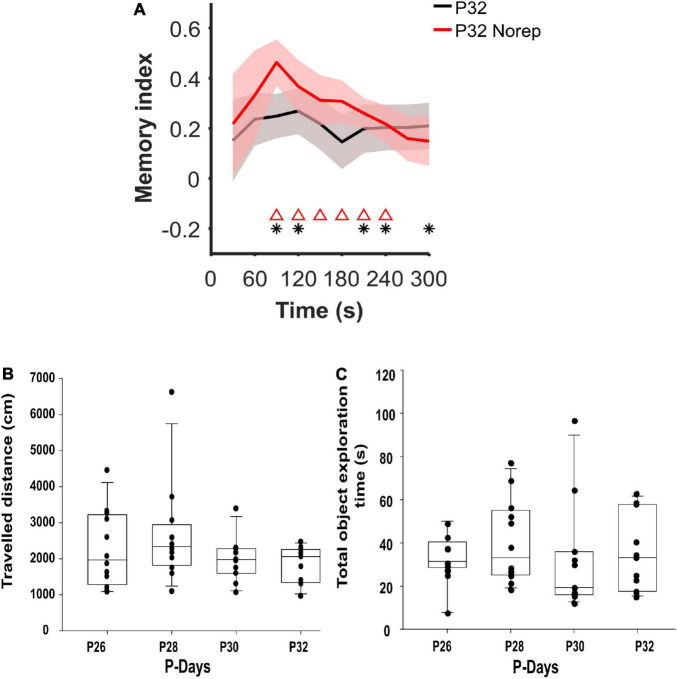
Memory emergence is not due to task overtraining. **(A)** Object memory index (Mean ± SEM) at P32 of repetitive exposure to the OPR task was compared with animals that performed the task without previous exposure (P32 NoRep). The memory indexes for the entire task duration (5 min, 30 s/bin) for P32 (black line) and P32 NoRep group (red line) indicated significant differences to the chance level (*p* < 0.0420), two-sided Wilcoxon-signed-rank for P32 (dark asterisk) and *p* < 0.0195 for P32 NoRep (red triangles). Groups P32 and P32 NoRep showed no significant differences (*p* > 0.0569, two-sided Wilcoxon rank-sum test). **(B)** No differences between the traveled distance during the 5-min of memory encoding at different rat ages were found [*p* = 0.08, One Way Repeated Measures Analysis of Variance *F*_(3,11)_ = 2.479]. **(C)** No differences between the total object exploration time were found, [*p* = 0.609, Friedman repeated-measures ANOVA on ranks, *F*_(3,11)_ = 0.617]. The halfway mark indicates the median on each box, while the bottom and top edges indicate the 25th and 75th percentiles. P26 and P28, *n* = 12; P30 and P32, *n* = 11.

Our data suggest that hippocampal-dependent spatial memory emerges at P32. However, the previous exposure and experience with the OPR task from P26–P30 may accelerate the natural emergence of memory capacities in the rodent. An additional group of animals performed the OPR task for the first time at P32 (P32 NoRep group, *n* = 11) to rule out this possibility. The P32 NoRep group showed a positive and significant memory index with respect to chance from 1.5 to 3.5 min (*p* < 0.0186; red triangles) without differing from the original P32 group (*p* > 0.057); which showed a significant memory index value at 1.5, 2, 3.5, 4, and 5 min (*p* < 0.042; black asterisk; Figure 2A). Therefore, our data support the finding that hippocampal-dependent memory consolidation emerges at P32 regardless of early task reinforcement. In addition, to assess whether our results were due to other changes related to the age of the animals (e.g., motor skills), we quantified the total distance traveled by the rats in the maze during the 5-min of the recall phase and the full exploration time of both objects. The total traveled distance remained constant over the postnatal days [*p* = 0.08, *F*_(3,11)_ = 2.479; Figure 2B]. Furthermore, the total object exploration time during the 5-min of the recall phase was unchanged across postnatal days [*p* = 0.609, *F*_(3,11)_ = 0.617; Figure 2C], revealing that the onset of spatial memory at P32 was not due to any difference in motor activity.

### Changes in Sleep Oscillations Through Development

To establish a link between sleep oscillations and hippocampal-dependent memory consolidation during postnatal development, we evaluated the profile of SO, Spindles, and SWRs in postnatal rats ages P26–P42 during NREM sleep. We successfully recorded extracellular LFP from the cortex (MPtA: medial parietal association area) and the dorsal hippocampus (CA1) in four animals. After memory encoding, the LFP activity was recorded during the 3 h of the retention phase.

**FIGURE 3:**
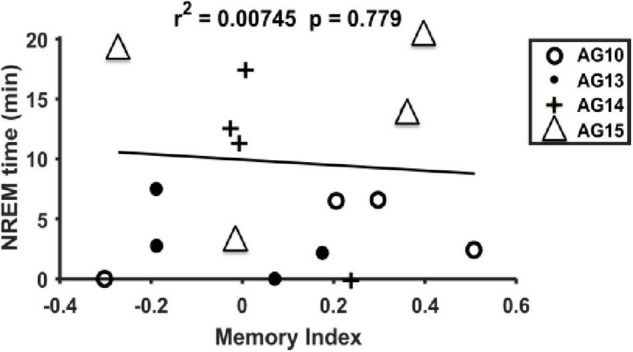
Linear regression between the time spent at NREM and the memory index. NREM time (min during 3 h of resting) vs. memory index for all day and animals (*n* = 4). No linear relationship was observed (*r*^2^ = 0.00745, *p* = 0.779).

Sleep in juvenile rats is highly variable and fragmented (Simasko and Mukherjee, 2009). Therefore, we first determined whether the amount of NREM sleep is a determinant factor of our OPR results. As seen in (Figure 3), there was no correlation between the amount of NREM sleep and the memory index (*r*^2^ = 0.00745; *p* = 0.779; see Supplementary Table 2 for detailed sleep measures).

**FIGURE 4:**
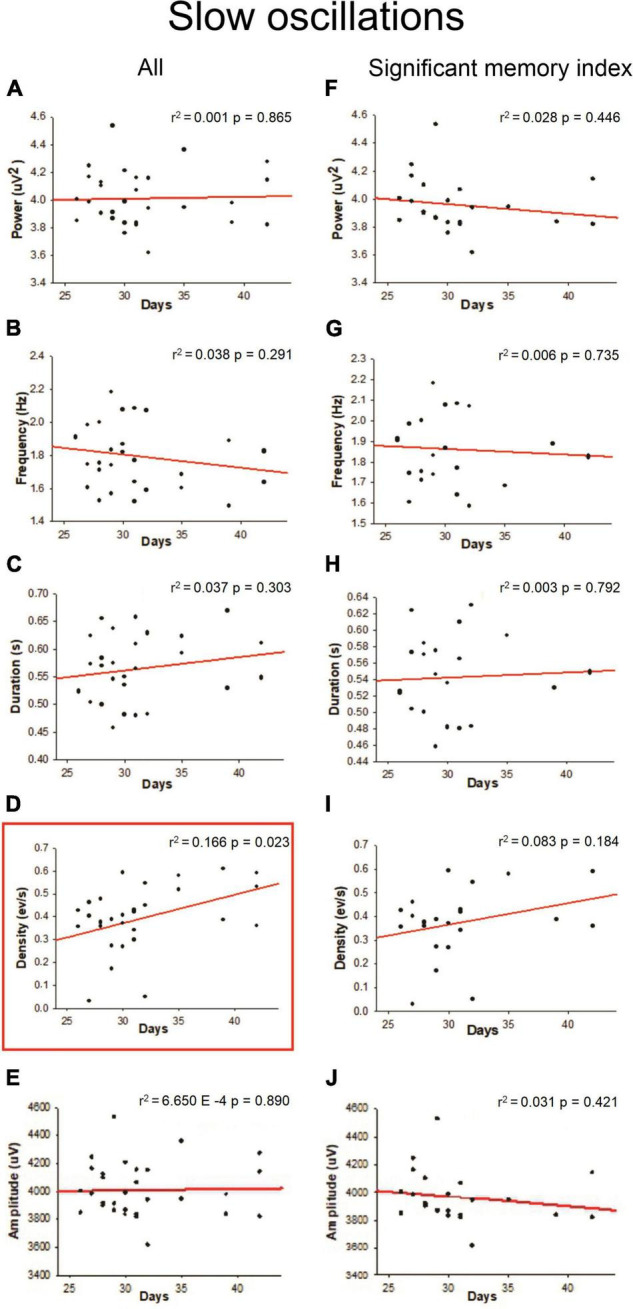
Linear regression between SO oscillation features with respect to developmental ages. For each postnatal day, the power **(A,F)**, frequency **(B,G)**, duration **(C,H)**, density **(D,I)**, and amplitude **(E,J)** were computed. The correlation analysis considered “All” animals (column on the left) or those with a “significant memory index” (column on the right). The *r*^2^ and *p*-value are indicated on each regression. Significant regression (*p* < 0.05) is enclosed in a square.

**FIGURE 5:**
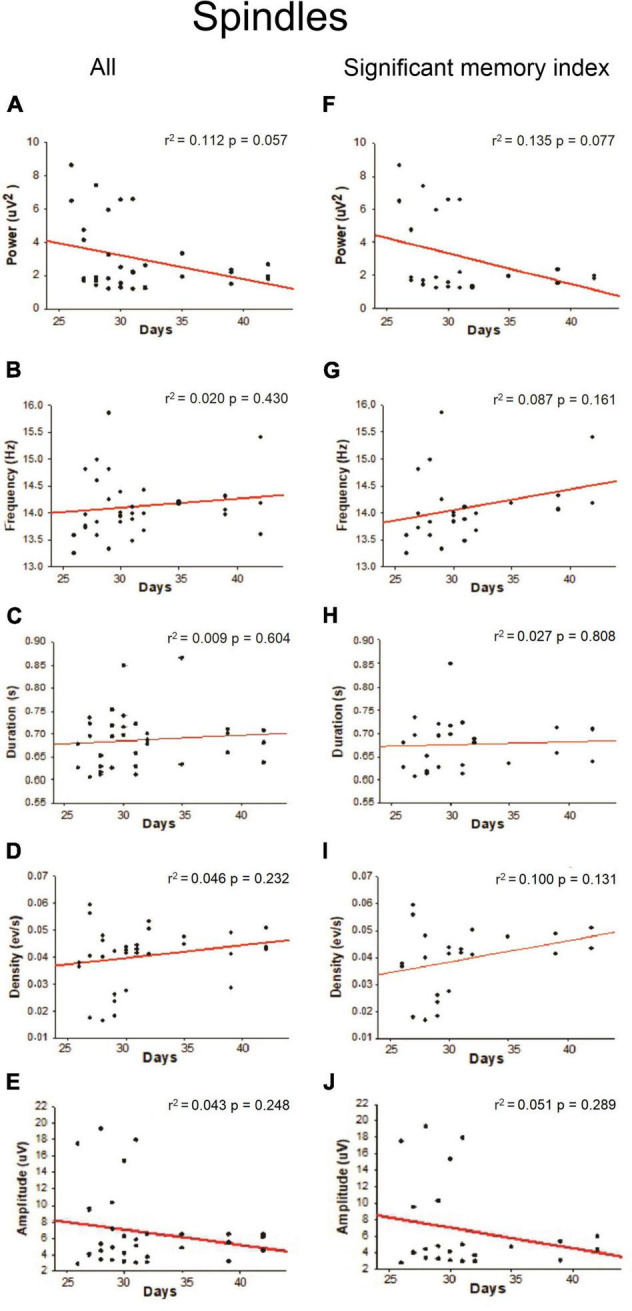
Linear regression between Spindles oscillation features with respect to postnatal days. For each postnatal day, the power **(A,F)**, frequency **(B,G)**, duration **(C,H)**, density **(D,I)**, and amplitude **(E,J)** were computed. The correlation analysis considered “All” animals (column on the left) or those with a “significant memory index” (column on the right). The *r*^2^ and *p*-value are indicated on each regression. No significant regressions were detected.

**FIGURE 6:**
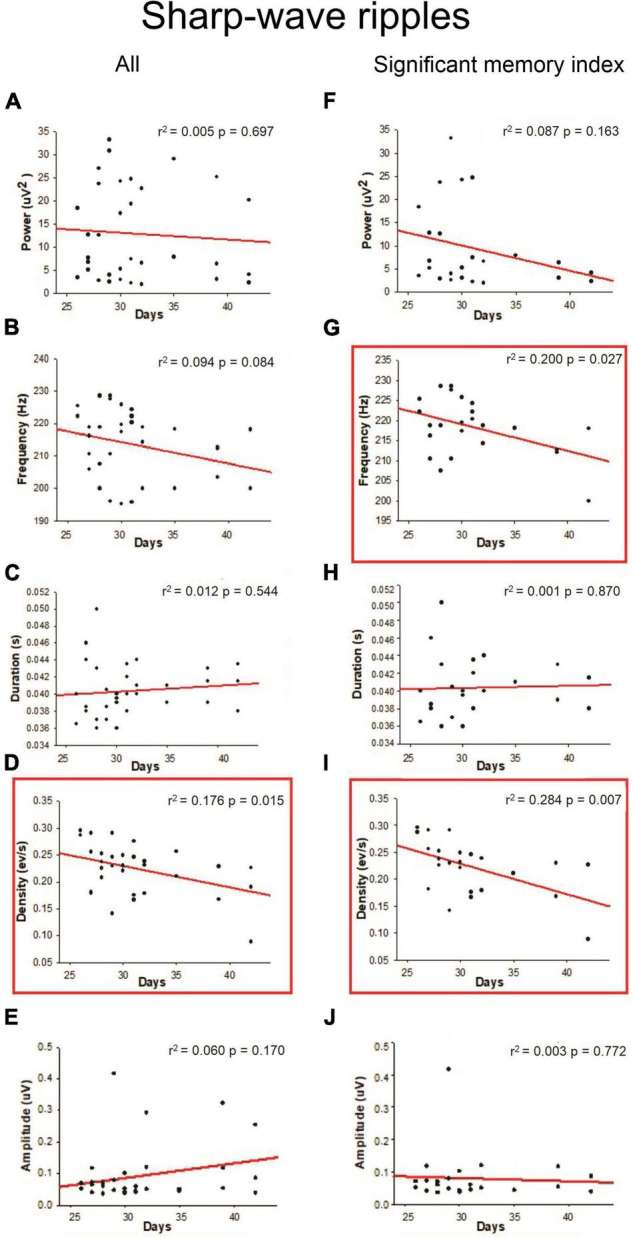
Linear regression between SWR oscillation features with respect to postnatal days. For each postnatal day, the power **(A,F)**, frequency **(B,G)**, duration **(C,H)**, density **(D,I)**, and amplitude **(E,J)** were computed. The correlation analysis considered “All” animals (column on the left) or those with a “significant memory index” (column on the right). The *r*^2^ and *p*-value are indicated on each regression. Significant regressions (*p* < 0.05) are enclosed in a square.

Slow-oscillations, Spindles and SWRs are characteristic events of the NREM sleep associated with memory consolidation, so we quantified the power, frequency, duration, density, and amplitude of those oscillations. As shown in Figure 4D, a positive linear relationship was found between density (SO events per min) during NREM sleep and age (*r*^2^ = 0.166; *p* = 0.023) when all the animals were considered; however, this correlation was not observed when only the animals with a significant memory index were taken into account (Figure 4I). Other SO features such as power frequency and duration were not correlated with age (Figures 4A–J). For Spindles, no correlation between different features of these oscillations and postnatal days was found (Figures 5A–J) when all animals were considered. For SWRs, we found that the SWR frequency decreased as a function of age (Figure 6G; *r*^2^ = 0.176; *p* = 0.015), but when only for animals with a significant memory index. A negative correlation between the density of SWRs and age was found for all animals and when only considering animals with a significant memory index (Figures 6D–I). Other features of SWRs were not correlated with age (Figures 6A–J).

### Hippocampal-Cortical Oscillatory Coupling Through Development

**FIGURE 7:**
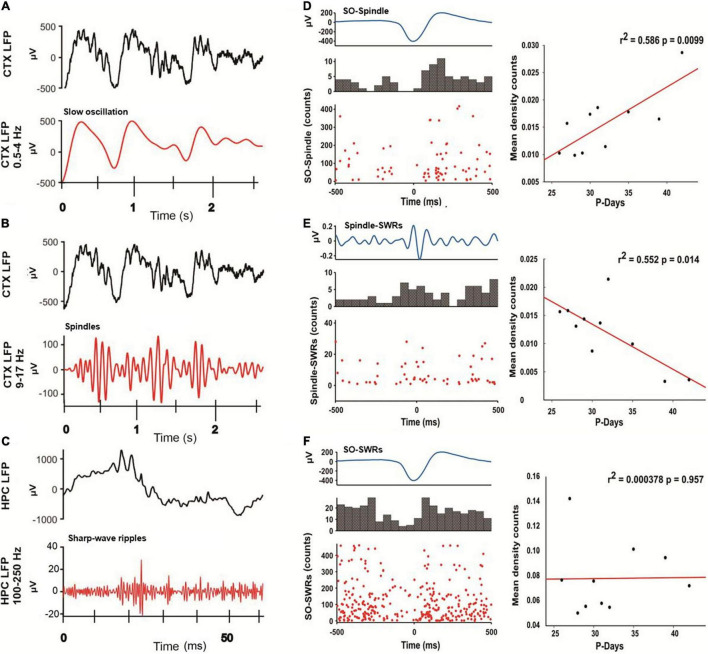
Temporal coupling between sleep-oscillations across postnatal ages. On the left panel, representative examples of LFP signals obtained from the cortex (CTX) and hippocampus (HPC) are indicated to illustrate the three oscillations detected in this present work. **(A)** Raw CTX-LFP in black, and its respective filtered signal in red (0.5–4 Hz) for SO events detection. **(B)** Raw CTX-LFP in black and respective filtered signal (9–17 Hz) in red for Spindle events detection. **(C)** Raw HPC-LFP in black and respective filtered signal (100–250 Hz) in red for a detected SWR. **(D)** Representative example of SO-Spindle coupling. On top, SO grand average waveform across all postnatal days. The SO down-state was centered at 0 lag. In the middle, a cross-correlation histogram showing the number of counts of Spindles temporal coupling events related to the SO down-state (± 500 ms). A raster plot shows all the Spindle-SO counts in the lower panel. Linear regression (panel on the right) among the mean density (counts/min) with respect to postnatal days. There is a positive correlation between SO and Spindles coupled events across postnatal days (*n* = 4; *r*^2^ = 0.586 and *p* = 0.0099). **(E)** A representative example of Spindle-SWR cross-correlation analysis as described before on top Spindle grand average waveform (Spindle peak centered at 0 lag). In the middle, a cross-correlation histogram shows the number of SWR counts related to the Spindle’s peak (± 500 ms), and the lower panel is a raster plot showing all the sharp-wave ripples events coupled to the Spindle’s peak. On the right, a negative relationship between the temporal coupling of Spindles and SWR across days was found (*r*^2^ = 0.0552, *p* = 0.014). **(F)** The same analysis was conducted for SO and SWR coupling events across days. The linear regression (panel on the right) indicates no linear relationship between those oscillatory events over time (*r*^2^ = 0.000378, *p* = 0.957).

To test the idea of the active model of memory consolidation (Marr, 1971; Buzsáki, 1989; McClelland et al., 1995; Frankland and Bontempi, 2005; Rasch and Born, 2007) during the development, we conducted a cross-correlation analysis between SO, Spindle, and SWR events during NREM sleep and postnatal age. As previously reported, the alignment between Spindles and the ascending phase of the SO may improve the consolidation of hippocampal-dependent memories (Latchoumane et al., 2017). We quantified the number of Spindle events within ± 500 ms of the SO for each NREM epoch (3,587 events) from all animals (*n* = 4), Figure 7. We observed a temporal coupling between SO and Spindles, where Spindles were nested in the SO up-phase (Figure 7D, left), consistent with previous report (Latchoumane et al., 2017; Hahn et al., 2020). Notably, we observed that the average number of coupled SO-Spindle events per day had a positive linear relationship with age (*r*^2^ = 0.586; *p* = 0.0099, Figure 7A, on the right), suggesting that SO-Spindle coupling strengthens during development.

As previously reported, the disruption of Spindles and SWR coupling is critical for spatial memory (Novitskaya et al., 2016); therefore, we quantified the SWR events whitin ± 500 ms of the peak of the Spindle (Figure 7E, left). We found that the average Spindle-SWR coupled events decreased with age (*r*^2^ = 0.552; *p* = 0.014, Figure 7E right). Additionally, we analyzed SWR events coupled to SO using the down-state of SO as 0 lag, and we found no relationship between the average SO-SWR and animal age (*r*^2^ = 0.000378; *p* = 0.957; Figure 7F). These findings could indicate that although SWR is driven by SO events (Mölle et al., 2006), the temporal coupling between those events during development may not be critical for memory consolidation.

**FIGURE 8:**
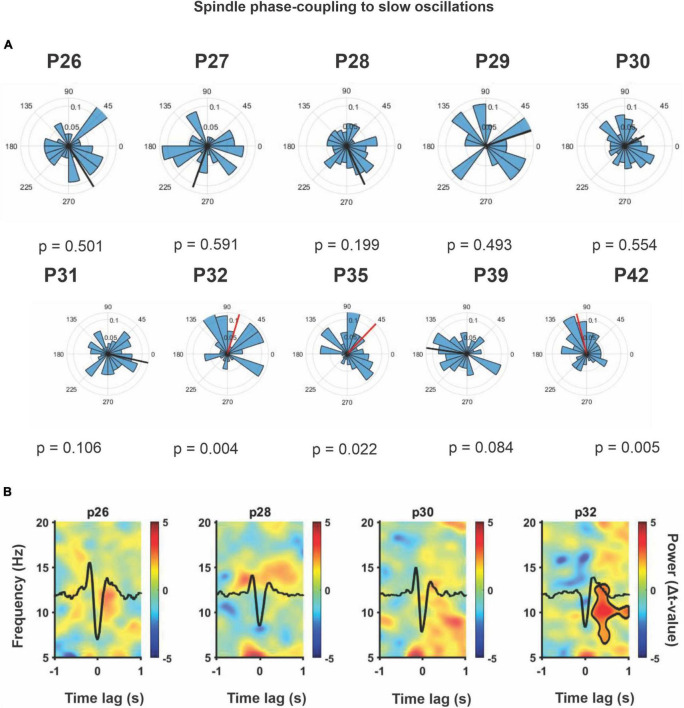
Spindle phase-coupling to slow oscillations. **(A)** Polar histograms across the postnatal day from P26 to P42 (*n* = 4). The resulting vector represents the mean direction and length for each case. The threshold for significant directionality was set at *p* = 0.05 (Rayleigh’s test for non-uniformity), significant is shown in red, otherwise in black. The 90° corresponds to the peak of the SO event (up-state) and 270° to the SO-trough (down-state). A significant directionality was detected at P32, P35, and P42. Spindle showed a preferent coupling distribution with the up-state of the SO (*p* = 0.022 or lower). **(B)** SO-triggered Spindle average spectrogram from cortical LFPs. SO down-state centered at 0 ± 1 s time lag. At P32, a significant power increase was observed in the Spindle frequency range (9–17 Hz) around the up-state of the SO. Statistical comparisons were performed through a non-parametric permutation test (MonteCarlo, *n* = 1000) using a cluster alpha of 0.02 and with a significance mask of *p* < 0.01. A significant cluster at P32 (*p* < 0.01) was indicated with a black contour.

**FIGURE 9:**
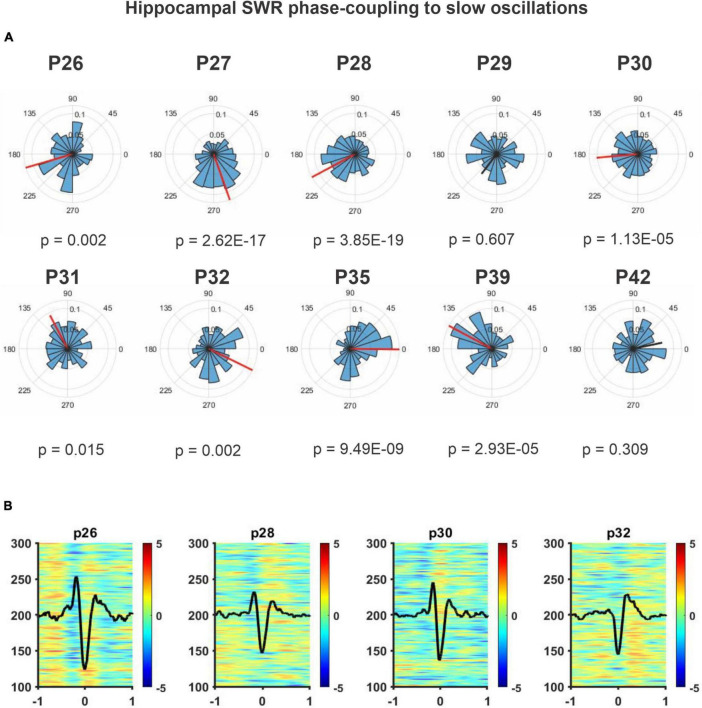
Hippocampal SWR phase-coupling to slow oscillations. **(A)** Polar histograms across the postnatal day from P26 to P42 (*n* = 4). The resulting vector represents the mean direction and length for each case. The threshold for significant directionality was set at *p* = 0.05 (Rayleigh’s test for non-uniformity), significant is shown in red, otherwise in black. The 90° corresponds to the peak of the SO event (up-state) and 270° to the SO-trough (down-state). Ripple events were coupled predominantly to the trough of the slow-wave (*p* < 0.015) across the postnatal days. Except for P42. **(B)** SO-triggered SWR average spectrogram from cortical LFPs. SO down-state centered at 0 ± 1 s. No changes in power in the SWR frequency range (100–200 Hz) were detected. Statistical comparisons were performed through a non-parametric permutation test (MonteCarlo, *n* = 1000) using a cluster alpha of 0.02 and with a significance mask of *p* < 0.01.

In addition, Spindle-SO phase coupling was measured from P26 to P42, Figure 8A, present polar histograms for each postnatal day, and we observed that by P32, a significant phase coupling is present. For this analysis, we considered postnatal days P26 to P42 to demonstrate that this coupling persisted in time even after P32, which is the critical day when memory capacities appeared. To complement this analysis (Figure 8B), we conducted a SO-triggered Spindle spectrogram during the OPR retention phase, from P26 to P32. We observed a significant increase in power at the Spindle frequency range (9–17 Hz) only at P32 during the upstate of the SO (black mask). The same analyses were performed for SWR and SO, and no developmental changes were found. The phase coupling between these two oscillations was present across the entire postnatal period (Figure 9A), and there was no significant increase in power detected in the range of SWR (100–300 Hz, Figure 9B). The number of the coupled Spindle-SWR was too low to support this same analysis and was not computed.

### Slow-Oscillations, Spindle and Sharp-Wave Ripples Parameters Correlate With Performance

**FIGURE 10:**
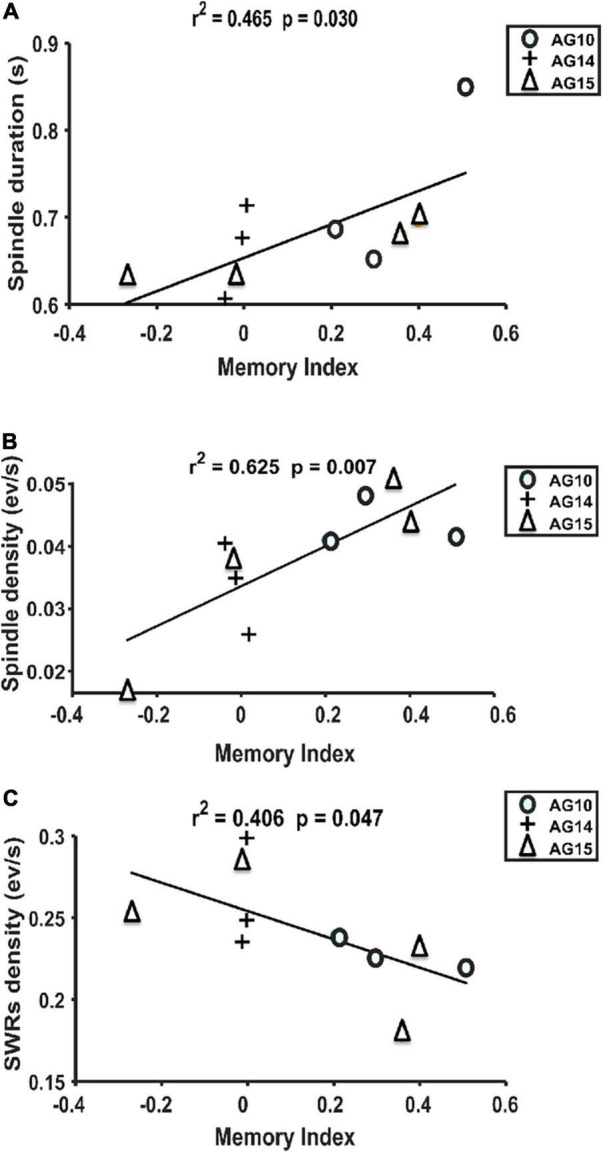
Linear regression between Spindles and SWR features concerning memory performance. **(A)** Spindle duration and memory performance positively correlate (*r*^2^ = 0.465 and *p* = 0.030). **(B)** Spindle density (events/min) respect to memory index gets also positively correlated (*r*^2^ = 0.625 and *p* = 0.007). **(C)** SWR density (events/min) and memory index show a negative correlation (*r*^2^ = 0.406 and *p* = 0.047). Each symbol represents different animals; only those that reached a significant memory index by P32 were included (*n* = 3). Two data points are missing because one animal did not fall asleep 1 day, and at P32, one animal lost the electrode recording implant.

Since some oscillatory features did not show a linear relationship with postnatal age, we wanted to explore whether those parameters could correlate with memory performance. We observed that rats with good performance in the memory task at P32 (*N* = 3) showed a positive and significant correlation between Spindle duration and memory index (*r*^2^ = 0.465; *p* = 0.030; Figure 10A), and Spindle density and memory index (*r*^2^ = 0.625; *p* = 0.007; Figure 10B). Conversely, a negative but significant relationship was observed between SWR density and memory performance (*r*^2^ = 0.406; *p* = 0.047; Figure 10C). No other correlation was found between subsequent oscillatory features and memory index (see Supplementary Table 3), suggesting that Spindles and SWRs may play a preponderant role in hippocampal-dependent memory consolidation through changes in either duration or density.

## Discussion

The present study investigated the relationship between sleep oscillations and hippocampal-dependent memory. Our main goal was to determine when hippocampal-dependent memory emerges during postnatal development (Westbrook et al., 2014; Ramsaran et al., 2016, 2019; Contreras et al., 2019; Farooq and Dragoi, 2019; Aggleton and Nelson, 2020) in concomitance with characterizing the sleep dynamics during the NREM-sleep-stage, which are thought to be essential for memory consolidation (Rasch and Born, 2013; Maingret et al., 2016; Latchoumane et al., 2017).

Our findings show that hippocampal-dependent memory emerges by P32 when rats successfully performed the OPR task, with memory indexes at adult levels independent of overtraining. At younger ages (P26, P28, and P30), rats showed no preference for either of the two objects, indicating poor memory performance. Although the memory index increases with age, the mechanism through which memory consolidation emerges could be associated with a complete maturation of the hippocampus and improves spatial-memory capacity. For example place-cells and grid cells develop until the 4–5 postnatal week, suggesting that memory consolidation would not be possible at younger ages (Farooq and Dragoi, 2019; Muessig et al., 2019). Similar results in hippocampal neuronal activity in an eyeblink conditioning paradigm have been demonstrated to depend on postnatal development (Goldsberry et al., 2015).

Consistent with our findings, hippocampal-dependent memories have long been thought to emerge late after the second postnatal week, although the ages have varied from P17 until as late as P38 (Westbrook et al., 2014; Ramsaran et al., 2016, 2019; Contreras et al., 2019). In younger animals, expression of hippocampal-dependent memory (P17–P21) was achieved using short-retention times (5 min- 1 h) and proximal cues as guidance (Westbrook et al., 2014; Ramsaran et al., 2019; Cruz-Sanchez et al., 2020). On the other hand, the late onset of allocentric memory reported at P38 by Contreras et al. (2019) could be explained because they did not look at ages younger than P31 and did not consider sleep as a contributing factor. Along with previous work, we could infer that our results fit into the category of allocentric hippocampal-dependent memory emergence; however, one potential problem was that juvenile rats might show hippocampal-dependent memory consolidation at a younger age due to overtraining. Since naïve rats at P32 did not differ from those animals that performed the task several times before P32, it suggests that there were no effects of overtraining on the emergence of memory capacities. In addition, differences due to motor skills, fatigue, anxiety, or lack of motivation were ruled out since the distance traveled and the total exploration time of the objects were the same across developmental days. This is the first report where the entire temporal window (P26–P32) has been explored using long retention times (3 h), allowing for sleep, distal cues as spatial guidance, and 24 h spacing between successive exposures to the OPR task.

Memory consolidation involves a finned-tuned hippocampal-cortical dialog between SO, Spindles, and SWRs (Buzsáki, 1989; Maingret et al., 2016; Latchoumane et al., 2017). SO commands the hierarchical nesting of thalamocortical Spindles and SWR, enabling the conversion of labile hippocampal memory traces to long-term cortical memory storage (Girardeau et al., 2009; Maingret et al., 2016; Niethard et al., 2018; Silversmith et al., 2020). However, how this coupling manifests along with development is still poorly understood. We first assessed the relationship of each independent oscillation parameter (i.e., power, duration, frequency, amplitude, and density) with age. Second, we examined the relationship between oscillation parameters and the memory index across ages. Finally, we evaluated the temporal coupling between SO, Spindles, and SWRs and looked at the relationship of phase coupling and density with the postnatal days and memory index over the range of postnatal age. We found a greater density in SO events and SO-Spindles temporal coupling along days.

Although the electrophysiological findings of this study are promising, they must be taken cautiously due to the small sample size (*n* = 4 in total; *n* = 3 for rats who showed learning). Some of the correlations between oscillation features were strong, but others may have been found with a larger sample size which was not possible in the current study. Hopefully, future research will replicate the present findings and potentially reveal more relationships between sleep oscillations and learning during development.

The precise temporal coordination of slow oscillations and sleep Spindles is a fundamental mechanism of sleep-dependent memory consolidation (Klinzing et al., 2016; Latchoumane et al., 2017). Recent studies in humans showed that the strength of coupling between SO and Spindles increases during the transition from infancy to adolescence, becoming a critical stage for memory formation (Hahn et al., 2020). Our results in rodents agree with that observation, suggesting that as rodents develop their spatial-memory capacities, SO and Spindles become more synchronized, supporting this process.

We also found that SWR density, frequency, and Spindle-SWR coupling diminish with age. SWR is essential for memory consolidation (Girardeau et al., 2009), emerging among P14–P20 (Buhl and Buzsáki, 2005). Previous studies have shown that age was associated with decreased density and frequency of SWR (Wiegand et al., 2016); however, it should be noted that the developmental period they used is much different than the period used in the current study. Another possibility is that we see an effect of overtraining. Even if we did not see an impact on behavior, there could be differences at the electrophysiological level. For example, repeated memories are more prone to becoming less dependent on the hippocampus and more on the cortex (Takashima et al., 2009). Thus, we could see reduced hippocampal activity when comparing tasks with repetition vs. single exposure. An alternative possibility is that even if the density of Spindle-SWR coupled events decreases, the few synchronized events occurring are enough to promote memory consolidation.

It is possible that a decrease in the density of the SWR, and their coupling to Spindles, is a characteristic change during this developmental period. More important than the density of coupled events in the SO up-state, particular Spindle or SWR features during coupling must be considered, such as amplitude, phase, or even neural activity. Furthermore, when we explored the phase coupling between Spindles and SO, we found that Spindles are aligned to the up-state of the SO by P32. Also, an increase in power at the Spindle frequency appears in the up-state portion of the SO. This power increase was not observed at the younger postnatal ages. The rise in power fits with slow Spindles (9–12 Hz) described by Mölle et al. (2011). However, previous studies in humans have described that slow Spindles are usually located in the up-to-down portion of the SO and fast Spindles (12–15 Hz) in the up-state of the SO (Andrillon et al., 2011; Klinzing et al., 2016). Further analysis needs to be conducted to determine how these two Spindles populations change through development as well as their relationship to SO. Nevertheless, our results at least suggest that slow Spindles are relevant for the early stages of the hippocampal-dependent memory process.

Human studies have shown a higher density of sleep-Spindles after performing a declarative memory task (Gais et al., 2002; Eschenko et al., 2006). In the current study, we found that only the animals that performed better in the OPR recall phase had Spindles of higher density and longer duration but did not observe any reliable developmental change in the density or duration of Spindles. This finding might indicate that the increase in Spindle density observed after OPR is related to memory performance more than it is related to changes associated with development. In contrast to Spindles, we observed that a lower density of SWRs was correlated with better performance, suggesting that fewer but more synchronized SWRs could be relevant for the memory consolidation process at this stage of development.

A relevant issue that arises from this study is the relationship between the cortical region chosen and the SO, Spindles, and SWR temporal coordination. Even though SO oscillations are a global phenomenon occurring across the entire neocortex and other subcortical structures (Neske, 2016), growing evidence in rodents and humans has demonstrated that SO are not synchronized across the whole neocortex. Specifically, they could even be locally distributed in particular cortical regions (Massimini et al., 2004; Vyazovskiy et al., 2011), which has also been observed for Spindles (Andrillon et al., 2011; Piantoni et al., 2017). This observation raises the question of how well activity in the hippocampus and MPTa get synchronized. The parietal cortex has been widely associated with visuospatial processing; however, its input-output connectivity with respect to the memory consolidation process has been poorly described.

There are several possible neuroanatomical pathways that could be proposed. First, CA1 sends projections to several cortical and subcortical regions, including temporal areas, facilitating direct interaction between the hippocampus and temporal cortex (Cenquizca and Swanson, 2007). Second, the hippocampus sends projections to the deep layer of the entorhinal cortex, which sends projections to the majority of the neocortex (Swanson and Cowan, 1977). Finally, the dysgranular retrosplenial cortex may mediate the coordination between the medial parietal cortex and hippocampus (Wilber et al., 2015). Independent of which pathway is most relevant to the results presented here, this is an exciting issue that will be addressed by future research.

In conclusion, our results demonstrate that hippocampal-dependent memory emerges at P32 independently of early overtraining. Sleep-oscillation parameters and phase coupling between oscillations are still changing, with some parameters showing a better fit to memory performance and others relating to maturational processes. These findings help us build a framework to study how “the active system mechanism” and “the active system consolidation theory” fit with the postnatal emergence of hippocampal-dependent memory. These results are important for understanding how the memory consolidation process described in adult animals works within the developing organism by combining behavioral and *in vivo* electrophysiological approaches, thereby giving new insight into the underlying mechanism of memory consolidation during sleep.

## Data Availability Statement

The raw data supporting the conclusions of this article will be made available by the authors, without undue reservation.

## Ethics Statement

The animal study was reviewed and approved by the Biosafety and Ethical Committee of the Universidad de Chile (CBA #1108, FMUCH) and Pontificia Universidad Católica de Chile (CEC-CAA ID:180430001).

## Author Contributions

MG-P: data acquisition and curing, analysis of behavioral, electrophysiological, and histological data, initial writing, and manuscript editing. MIr: electrophysiology data analysis. VT: behavioral data analysis. TB: histology analyses. MIn: conceptualization of the work, experimental design, and initial data collection. PM and JV: conceptualization, funding, project administration, writing, and manuscript editing. All authors read and approved the manuscript.

## Conflict of Interest

The authors declare that the research was conducted in the absence of any commercial or financial relationships that could be construed as a potential conflict of interest.

## Publisher’s Note

All claims expressed in this article are solely those of the authors and do not necessarily represent those of their affiliated organizations, or those of the publisher, the editors and the reviewers. Any product that may be evaluated in this article, or claim that may be made by its manufacturer, is not guaranteed or endorsed by the publisher.
