# Supplementary material for: Cortico-Hippocampal Oscillations Are Associated With the Developmental Onset of Hippocampal-Dependent Memory
Source: Front Neurosci. 2022 Jun 23;16:891523. doi: 10.3389/fnins.2022.891523 (PMC9260104; doi:10.3389/fnins.2022.891523)
Supplement: Supplementary file 1 [file Image_1.pdf]

Supplementary Material

Supplementary Figures

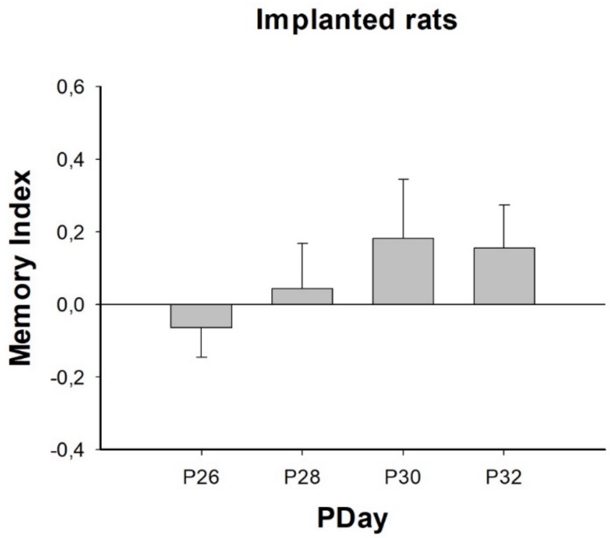

Supplementary Figure 1. Memory performance in the OPR task for the four rats used in the electrophysiological analysis. These four rats come from the 12 animals used for behavioral analysis described in the main text. Notice similar performance compared with the average performance.

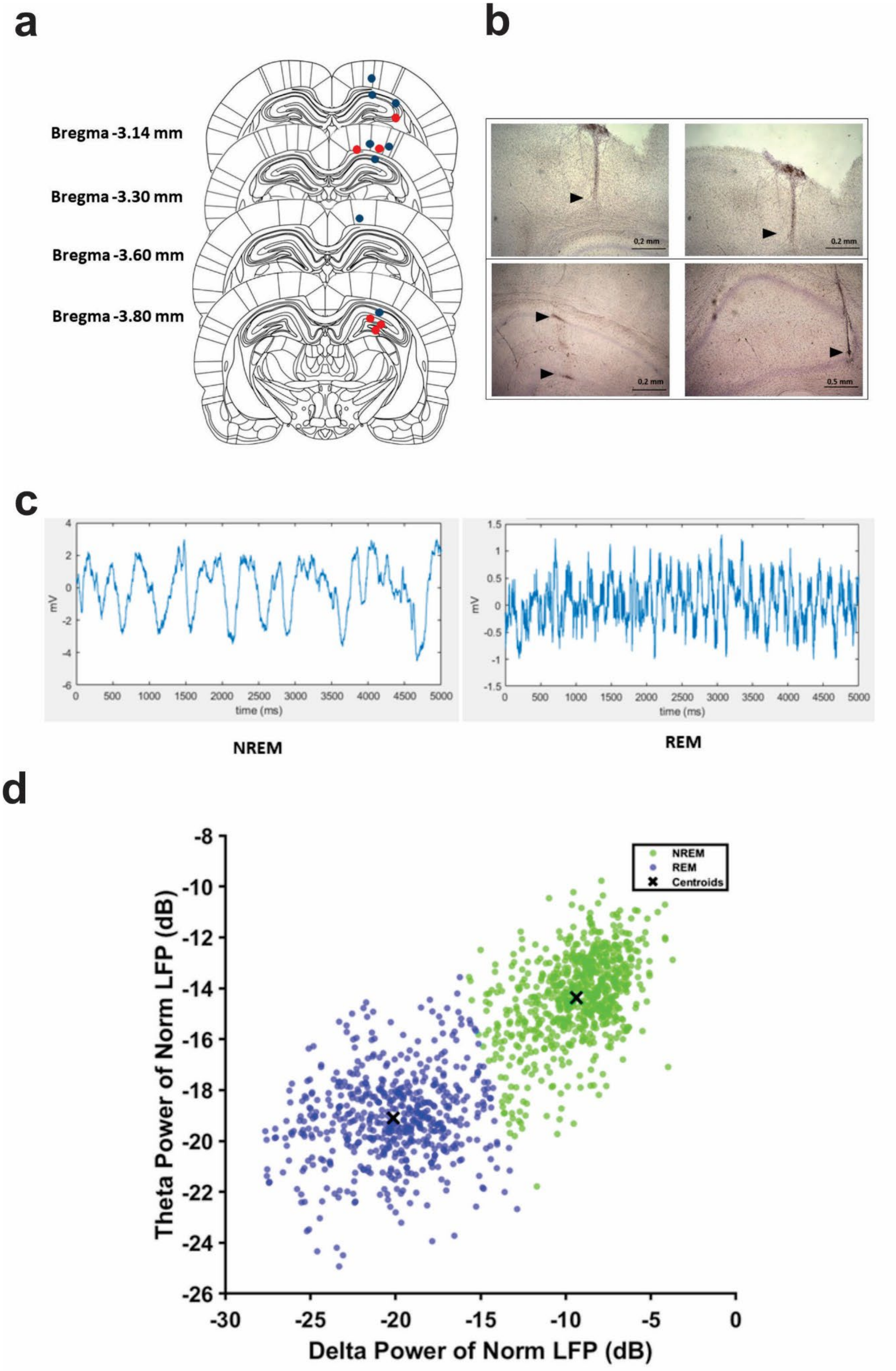
a
b
Bregma -3.14 mm
Bregma -3.30 mm
Bregma -3.60 mm
Bregma -3.80 mm
0,2 mm
0,2 mm
0,2 mm
0,5 mm
c
mV
time (ms)
NREM
REM
d
NREM
REM
Centroids
Theta Power of Norm LFP (dB)
Delta Power of Norm LFP (dB)

Supplementary Figure 2. Supplementary Fig. 1. a) Reconstruction of electrodes tip position at the recorded animals, dot in blue indicated the electrodes used for cortical and hippocampal electrophysiological analysis, while the red ones reconstructed tip electrodes were not included in the analysis. The tip positions of the electrodes were reconstructed by inspection of the Nissl-stained brain section. Arrows indicate an example of electrodes track and tips in b). In c) typical examples of LFP signals considered NREM and REM sleep, respectively. d) Clustering NREM and REM sleep epochs, using the delta/theta power spectrum relationship.
