# Supplementary material for: Cortico-Hippocampal Oscillations Are Associated With the Developmental Onset of Hippocampal-Dependent Memory
Source: Front Neurosci. 2022 Jun 23;16:891523. doi: 10.3389/fnins.2022.891523 (PMC9260104; doi:10.3389/fnins.2022.891523)
Supplement: Supplementary file 2 [file Data_Sheet_1.docx]

Supplementary Material

# Supplementary Tables

| **Animal ID** | | | | **Pday** |
| --- | --- | --- | --- | --- |
| **AG10** | **AG13** | **AG14** | **AG15** |  |
| NSD | NSD | 0.29577465 | 0.286486486 | 26 |
| 0.22531646 | 0.20769231 | 0.23733333 | 0.252173913 | 28 |
| 0.22068966 | 0.23030303 | 0.2490566 | 0.23127572 | 30 |
| 0.23846154 | 0.23111111 | NSD | 0.178443114 | 32 |

**Supplementary Table 1:** Each animal's ripple density (ripple/sec) across different days. NSD no detected sleep.

| **Pday** | **Awake time** | **REM time** | **NREM time** | **Awake (%)** | **REM (%)** | **NREM(%)** |
| --- | --- | --- | --- | --- | --- | --- |
| **P26** | 9610.000 | 742.500 | 447.500 | 88.981 | 6.875 | 4.144 |
| **P27** | 9442.500 | 843.750 | 513.750 | 87.431 | 7.813 | 4.757 |
| **P28** | 8073.750 | 1457.500 | 1268.750 | 74.757 | 13.495 | 11.748 |
| **P29** | 9937.500 | 493.750 | 368.750 | 92.014 | 4.572 | 3.414 |
| **P30** | 8453.750 | 1023.750 | 1322.500 | 78.275 | 9.479 | 12.245 |
| **P31** | 9478.750 | 738.750 | 582.500 | 87.766 | 6.840 | 5.394 |
| **P32** | 9623.333 | 618.333 | 558.333 | 89.105 | 5.725 | 5.170 |
| **P35** | 9740.000 | 377.500 | 682.500 | 90.185 | 3.495 | 6.319 |
| **P39** | 9873.333 | 528.333 | 398.333 | 91.420 | 4.892 | 3.688 |
| **P42** | 9570.000 | 585.000 | 645.000 | 88.611 | 5.417 | 5.972 |

**Supplementary Table 2:** Detailed time (sec) and percentage of time during wake/sleep cycle across different postnatal days.

|  | **Memory Index** | **Power** | **Frequency** | **Duration** | **Density** |
| --- | --- | --- | --- | --- | --- |
| **All rats** | **SO** | r² = 0.132  p = 0.204 | r² = 0.011  p = 0.736 | r² = 0.007  p = 0.779 | r² = 0.007  p = 0.781 |
|  | **Spindles** | r² = 0.150  p = 0.190 | r² = 0.011  p = 0.733 | r² = 0.166  p = 0.167 | r² = 0.186  p = 0.141 |
|  | **SWRs** | r² = 0.148  p = 0.194 | r² = 0.015  p = 0.664 | r² = 0.039  p = 0.519 | r² = 0.230  p = 0.097 |
| **Rat with significant memory index** | **SO** | r² = 0.013  p = 0.738 | r² = 0.003  p = 0.884 | r² = 0.005  p = 0.849 | r² = 0.001  p = 0.929 |
|  | **Spindles** | r² = 0.295  p = 0.105 | r² = 0.159  p = 0.254 | **r² = 0.465**  **p = 0.030** | **r² = 0.625**  **p = 0.007** |
|  | **SWRs** | r² = 0.116  p = 0.335 | r² = 0.278  p = 0.117 | r² = 0.056  p = 0.509 | **r² = 0.406**  **p = 0.047** |

**Supplementary Table 3:** Different oscillation parameters were correlated with behavioral performance. The power. frequency. duration. and density were quantified for each oscillation. The analysis was conducted using all rats or only those with a significant memory index by P32. For each linear regression. the r^2^ and p-value are indicated. Significant p-values (p < 0.05) are in bold.
